# Convolutional neural network for automated classification of osteonecrosis and related mandibular trabecular patterns

**DOI:** 10.1016/j.bonr.2022.101632

**Published:** 2022-10-29

**Authors:** Soroush Baseri Saadi, Catalina Moreno-Rabié, Tim van den Wyngaert, Reinhilde Jacobs

**Affiliations:** a OMFS-IMPATH Research Group, Department of Imaging and Pathology, Faculty of Medicine, University of Leuven, Leuven, Belgium; b Department of Oral and Maxillofacial Surgery, University Hospitals Leuven, Leuven, Belgium; c Department of Nuclear Medicine, Antwerp University Hospital, Edegem, Belgium; d Faculty of Medicine and Health Sciences, University of Antwerp, Antwerp, Belgium; e Department of Dental Medicine, Karolinska Institutet, Stockholm, Sweden

**Keywords:** Osteonecrosis, Panoramic radiography, Diagnostic imaging, Artificial intelligence, Convolutional neural network

## Abstract

**Objective:**

The present study aimed to develop and validate a tool for the automated classification of normal, affected, and osteonecrosis mandibular trabecular bone patterns in panoramic radiographs using convolutional neural networks (CNNs).

**Methods:**

A dataset of 402 panoramic images from 376 patients was selected, comprising 112 control radiographs from healthy patients and 290 images from patients treated with antiresorptive drugs (ARD). The latter was subdivided in 70 radiographs showing thickening of the lamina dura, 128 with abnormal bone patterns, and 92 images of clinically diagnosed osteonecrosis of the jaw (ONJ). Four pre-trained CNNs were fined-tuned and customized to detect and classify the different bone patterns. The best performing network was selected to develop the classification tool. The output was arranged as a colour-coded risk index showing the category and their odds. Classification performance of the networks was assessed through evaluation metrics, receiver operating characteristic curves (ROC), and a confusion matrix. Furthermore, Gradient-weighted Class Activation Mapping (Grad-CAM) was employed to visualise class-discriminative regions.

**Results:**

All networks correctly detected and classified the mandibular bone patterns with optimal performance metrics. InceptionResNetV2 showed the best results with an accuracy of 96 %, precision, recall and F1-score of 93 %, and a specificity of 98 %. Overall, most misclassifications occurred between normal and abnormal trabecular bone patterns.

**Conclusion:**

CNNs offer reliable potentials for automatic classification of abnormalities in the mandibular trabecular bone pattern in panoramic radiographs of antiresorptive treated patients.

**Clinical significance:**

A novel method that supports clinical decision making by identifying sites at high risk for ONJ.

## 1 Introduction

The radiographic appearance of the mandibular trabecular bone pattern is a recurring topic of interest in dental research due to its direct impact on the prognosis of bone tissue-related treatments, such as dental implant placement (Nicolielo et al., 2020). When beginning with the bone pattern assessment, panoramic radiographs are a useful and widely available diagnostic tool (Pachêco-Pereira et al., 2019; Taguchi et al., 1997). They allow the identification of bony changes, which are caused by different reasons, including systemic diseases like osteoporosis or diabetes (Pachêco-Pereira et al., 2019), condensing osteitis (Lahoud et al., 2022), and the use of antiresorptive drugs (ARDs), namely bisphosphonates and denosumab (Moreno-Rabié et al., 2020).

ARDs are effective medications used to manage oncological conditions secondary to bone metastases and osteoporosis-related fractures (Ruggiero et al., 2022). Their mechanism of action alters the bone resorption-apposition cycle by impeding osteoclast activity through different pathways (Baron et al., 2011), thus favouring bone apposition. Consequently, the use of these drugs has been associated with radiographic findings on panoramic radiographs (Moreno-Rabié et al., 2020; Ruggiero et al., 2022) and the development of a side effect known as Medication-Related Osteonecrosis of the Jaws (MRONJ) (Marx, 2003; Ruggiero et al., 2022). The latter can be clinically identified as exposed bone in the oral cavity present for more than eight weeks in patients treated with ARD (Ruggiero et al., 2014, Ruggiero et al., 2022).

Patients receiving ARD treatment and without bone exposure may show in their panoramic images, osteosclerosis, thickening of the lamina dura and of the mandibular cortical, osteolytic areas, persistence of the extraction socket, and widening of the periodontal ligament space (Moreno-Rabié et al., 2020). On the other hand, MRONJ lesions show sclerosis, lytic changes, periosteal reaction, and sequestrum formation (Walton et al., 2019). These radiographic findings are important to identify, specially before the onset of osteonecrosis since some may act as predisposing factors for its occurrence. In fact, heterogeneous (Gaêta-Araujo et al., 2021) and sclerotic trabecular bone patterns (Kubo et al., 2018; Moreno-Rabié et al., 2022) have been identified as risk factors for MRONJ.

It is in the identification of these radiographic findings that clinicians could benefit from an objective and automated approach. The role of deep learning, specifically with convolutional neural networks (CNNs), has gained great importance in the classification, detection, and segmentation of objects of interest in medical imaging (Yamashita et al., 2018), showing promising results in dental applications both with two- and three-dimensional images. For instance, CNNs have been applied to automatically detect and segment teeth (Kuwada et al., 2020; Leite et al., 2021; Vranckx et al., 2020) and cystic lesions (Kwon et al., 2020) in panoramic radiographs. Moreover, examples of applications of CNNs in Cone-Beam Computed Tomography (CBCT) include, mandibular canal segmentation (Lahoud et al., 2022) and tooth segmentation and classification (Shaheen et al., 2021).

Based on the prior evidence, the main aim of this study is to develop and validate a tool for the automated classification of normal, affected, and osteonecrosis mandibular trabecular bone patterns in panoramic images using CNNs.

## 2 Material and methods

### 2.1 Study design and settings

The ethical committee of UZ/KU Leuven approved the elaboration of this retrospective cohort study (reference number: MP018766) and waived the need for informed consent. In addition, the World Medical Association Declaration of Helsinki and the standards of the Institutional Review Board were obeyed. To perform this study, panoramic radiographs were collected from patients treated in the department of oral and maxillofacial surgery at the University Hospitals of Leuven in Belgium.

### 2.2 Dataset

Panoramic radiographs were obtained from patients older than 18 years, treated with at least one administration of ARD, who fit into one of the following three groups, (1) showing thickening of the lamina dura (TLD), (2) abnormal bone pattern (ABP) such as bone sclerosis or persistence of the extraction socket, or (3) presenting with medication-related osteonecrosis of the jaw (MRONJ). Clinically, the first two patient groups had at the time of radiographic acquisition absence of bone exposure in the oral cavity, while the third group had a diagnosed MRONJ lesion with consequent clinical bone exposure (Ruggiero et al., 2014). In addition, a control group of healthy patients without ARD treatment, absence of maxillofacial pathologies, and with a normal bone pattern, was selected. These images were acquired with two different panoramic radiographic machines (Vista Pano S Ceph, Durr Dental, Bissingen-Bietigheim, Germany and Promax 2D, Planmeca, Helsinki, Finland), had a dimension of 2880 × 1504 pixels, and were anonymized at the time of export. Their indication was diagnosis and/or treatment planning for reasons other than the participation to this study.

A dataset of 402 panoramic images from 376 patients were selected. The mean age of the patients was 61 years (SD ± 18.6, range 25–94) in the control group, 64 years (SD ± 13.8, range 18–94) in the TLD, 69 years (SD ± 9.6, range 38–91) in the ABP, and 70 years (SD ± 10.3, range 48–91) in the MRONJ group. From the total images, 112 belonged to healthy control patients and 290 to patients treated with ARDs. The latter group was subdivided in 70 radiographs showing thickening of the lamina dura, 128 with abnormal bone pattern, and 92 images of clinically diagnosed osteonecrosis of the jaw.

Once all images were collected, mandibular regions of interest (ROIs) of 512 × 512 pixels were cropped using GIMP software (version 2.10.22, GIMP Development Team, CA, USA), resulting in 236 croppings from the control group, 126 with thickened lamina dura, 251 with abnormal bone pattern, and 131 cut-offs with osteonecrosis of the jaw. The image selection, cropping, and labelling was performed by a general dentist (CMR) and revised by a dentomaxillofacial radiologist (RJ) with 30 years of experience, serving as ground truth. The complete dataset was randomly divided into three sets using the python split-folders library (version 0.5.1, licensed from MIT, MA, USA). The same proportion of images from each group was assigned to each set, resulting in 536 images in the training set (70 % of the images of each group), 74 images in the validation set (10 %), to test the performance of the models during the training phase, and 134 images in the test set (20 %), used to evaluate the performance of the models by comparing the results with the ground truth data.

**Fig. 1:**
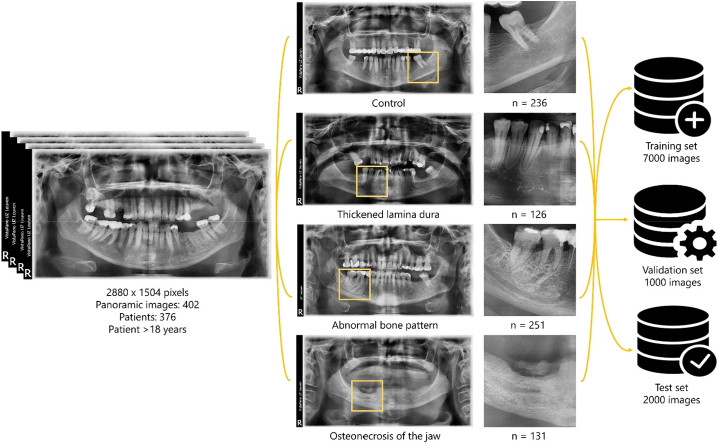
Workflow from image collection to the final number of images used for training, validation and testing of the algorithms. Once collected, the images were classified into different groups, areas of interest of 512 × 512 pixels were selected and augmentation techniques were performed until the final number of 10,000 images was reached.

Due to the limited dataset, data augmentation was performed using an open source Python library, Albumentations augmentation library (Buslaev et al., 2020). Transformations from pixel- to spatial-level were implemented to prevent overfitting and provide optimal results. Augmentation methods led to a dataset of 10,000 images. The networks were trained and validated with 7000 (70 % of dataset) and 1000 (10 % of dataset) images, respectively. The remaining 2000 images (20 % of dataset) were used as test set with 500 images obtained for each of the four classes. Fig. 1 shows the workflow until reaching the final dataset.

### 2.3 AI framework

Three popular very deep convolutional neural networks and one mobile design CNN architecture for resource constrained environments were selected to perform the classification task in the present study. The selected networks and their layer numbers were as follows, ResNet152V2 (*n* = 152) (He et al., 2016), InceptionResNetV2 (*n* = 164) (Szegedy et al., 2016), Densenet201 (*n* = 201) (Huang et al., 2017), and MobileNetV2 (*n* = 53) (Sandler et al., 2018).

The models were implemented through Keras library and its applications (Chollet and Others, 2015) using transfer learning methods, pre-designed models and pre-trained weights. The selected networks were designed based on a variation of deep residual learning principle (He et al., 2016) and pretrained with ImageNet database (Russakovsky et al., 2015).Once the models were extracted from Keras library, they were customized by replacing the classification head with a 512-unit dense layer, the ‘Sigmoid’ activation function using ‘He_uniform’ as the kernel initializer, a 25 % dropout layer, and a fully connected layer with ‘Softmax’ activation function and four outputs. The last modification aimed to homologate the number of classes in the dataset. All models' layers were set to be untrainable with the exception of the last customized layer. The datasets were read, pre-processed and resized to a resolution of 256 × 256 pixels using OpenCV (Bradski, 2000), Matplotlib (Hunter, 2007) and Numpy libraries (Harris et al., 2020). Then, they were normalized to a fixed range (0,1) for training, validation, and test phases. Training of the models was performed with categorical cross-entropy as the loss function, Adam's algorithm as the optimizer with an initial learning rate of 0.001, a batch size of 32, and a weight decay parameter of 2e-4. The models achieved convergence at different epochs by controlling the validation loss using early stopping with the patience rate of 30.

The best performing model was selected to develop a desktop classification tool. The tool was developed using PyQt5 library (version 5.15.7, Riverbank Computing Limited, Dorchester, UK), a set of cross-platform libraries in C++ that provides high-level application programming interfaces (APIs). The output was presented as a colour-coded clinical risk index to provide clinicians with a straightforward scale to determine the referral and treatment needs of individual patients undergoing treatment with ARD. The colour-coded classification indicated in green a normal trabecular bone pattern. Yellow showed thickening of the lamina dura as an indicator of bone changes induced by antiresorptive drugs with an initial advice for a cautionary surgical approach. Orange referred to an abnormal bone pattern attributed to the use of antiresorptive drugs and indicates potential negative bone remodelling with a warning to limit surgical trauma to that area. Finally, red would imply recognition of osteonecrosis of the mandible. The colour classification was accompanied by a relative probability for the region of interest.

Modelling was performed using Keras deep learning framework (version 2.10.0)(Chollet and Others, 2015), Tensorflow (version 2.10.0) and tensorflow-gpu (version 2.10.0) (Abadi et al., 2016) and implemented on an Intel(R) Xeon(R) W-2104 CPU@3.20GHz 3.19 GHz with 32.0 GB Ram, and a graphic card of NVIDIA Quadro P4000 GPU (NVIDIA Corporation, U.S.A) with a memory of 8 GB GDDR5.

### 2.4 Evaluation metrics

The following multiclass classification metrics (Grandini et al., 2020) were used to evaluate the performance of the CNN models on the test dataset:

• Accuracy: percentage of correctly classified images considering the whole sample.
  $$\mathrm{Accuracy}=\frac{\mathrm{TP}+\mathrm{TN}}{\mathrm{TP}+\mathrm{TN}+\mathrm{FP}+\mathrm{FN}}$$
• Precision: percentage of correctly classified positives from all assigned positives.
  $$\left(\mathrm{Precision}\right)=\frac{\mathrm{TP}}{\mathrm{TP}+\mathrm{FP}}$$
• Recall (Sensitivity): percentage of correctly classified positives from the ground truth.
  $$\left(\mathrm{Recall}\right)=\frac{\mathrm{TP}}{\mathrm{TP}+\mathrm{FN}}$$
• F1-score: weighted average between precision and recall in percentage.
  (F1-score)$=2∙\left(\;\frac{\mathrm{Precesion}∙\mathrm{Recall}}{\mathrm{Precision}+\mathrm{Recall}}\;\right)$
• Specificity: percentage of correctly classified negatives from the ground truth.
  $$\left(\mathrm{Specificity}\right)=\frac{\mathrm{TN}}{\mathrm{TN}+\mathrm{FP}}$$

where true positive (TP) indicates the correctly classified images among the different categories, true negative (TN), the number of images where the model correctly classified as not belonging to a group, false positive (FP), the number of the images where the network misclassified as belonging to a group, and false negative (FN), express the classifications where the model incorrectly classified an image as not belonging to a group, but it did belong. In other words, these values assess the ability of the system to classify the images properly.

The models were further assessed using tf-keras-vis library (version 0.8.2, MIT Licence, MA, USA) to implement the explainable artificial intelligence (XAI) elements, were a Gradient-weighted Class Activation Mapping (Grad-CAM) (Selvaraju et al., 2020) was obtained to provide a visual localization of class-discriminative regions. In addition, a Receiver Operating Characteristic (ROC) curve and confusion matrix were acquired with Scikit-learn library (Pedregosa et al., 2011) from each model to evaluate their classification performance.

### 2.5 Statistical analysis

RStudio version 4.0.4 (RStudio, Boston, MA US) was used to perform the statistical analysis. The metric values were tested for normality using Shapiro–Wilk test and visual inspection with a Q-Q Plot. Then, the Kruskal Wallis test was implemented to test the statistical significance of the accuracy, precision, F1-score, recall (sensitivity), and specificity between the models. A *p*-value ≤0.05 was considered statistically significant.

## 3 Results

The results of the evaluation metrics computed for each network on the test set are presented in Table 1. Overall, the best results were achieved by InceptionResNetV2 and MobileNetV2 with an accuracy of 96 %, while Densenet201 showed the lowest performance with 88 % of accuracy. Although, when performing the Kruskal Wallis test, the statistical computations demonstrated that there was no significant difference between the accuracy (*p* = 0.152), precision (*p* = 0.150), recall (*p* = 0.119), F1-score (*p* = 0.164), and specificity (*p* = 0.117) of the models. Given the slightly better classification metrics, InceptionResNetV2 was chosen as the CNN with which the classification model would be developed. An example of the interface can be seen in Fig. 6.

**Table 1:** Classification metrics used to evaluate the performance of the CNN models on the test dataset. These values were obtained by comparing the results of the classification with the ground truth (mean, 95 % CI).

Model	Parameters	Accuracy (%)	Precision (%)	F1-Score (%)	Recall (%) (sensitivity)	Specificity (%)
InceptionResNetV2	55.1 M	0.96 (0.93, 0.99)	0.93 (0.84, 1)	0.93 (0.87, 0.99)	0.93 (0.83,1)	0.98 (0.94, 1)
ResNet152V2	59.3 M	0.95 (0.92, 0.99)	0.91 (0.84, 0.98)	0.91 (0.82, 0.99)	0.91 (0.79, 1)	0.97 (0.94, 1)
Densenet201	18.8 M	0.88 (0.78, 0.98)	0.77 (0.55, 0.98)	0.76 (0.57, 0.94)	0.76 (0.55, 0.96)	0.92 (0.83, 1)
MobileNetV2	2.9 M	0.96 (0.93, 1)	0.92 (0.85, 0.99)	0.92 (0.84, 0.99)	0.92 (0.77, 1)	0.98 (0.97, 0.99)

**Fig. 2:**
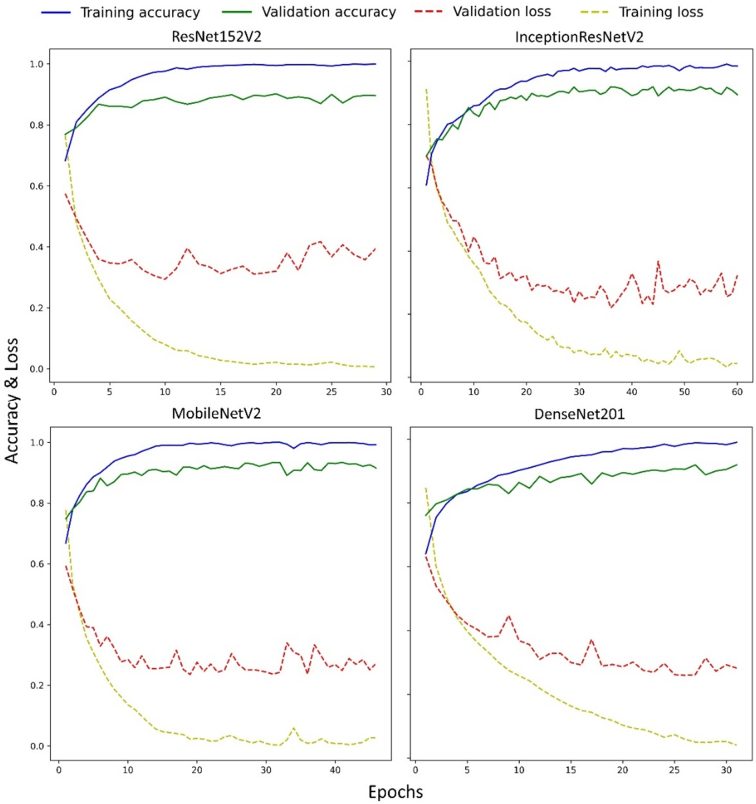
Training history of the networks depicting the loss and accuracy values of the models at different epochs during training and validation phases.

The training history of the models is presented in Fig. 2. The models' performance improved as the number of epochs increased. A slight overfitting was observed in the accuracy and loss values of the training and validation sets in each model. Moreover, convergence was achieved at different epochs, being in ResNet152V2 at 29, in InceptionResNetV2 at 60, in MobileNetV2 at 46, and in DenseNet201 at 31 epochs.

**Fig. 3:**
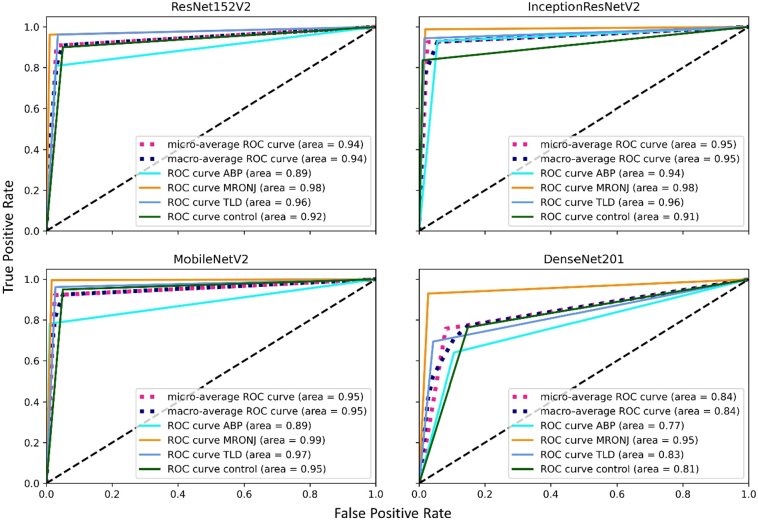
Receiver operating characteristic curves (ROC) of the models on the test dataset. The Area Under the Curve (AUC) for each class is shown as well as the micro and macro-averages across all classes.

**Fig. 4:**
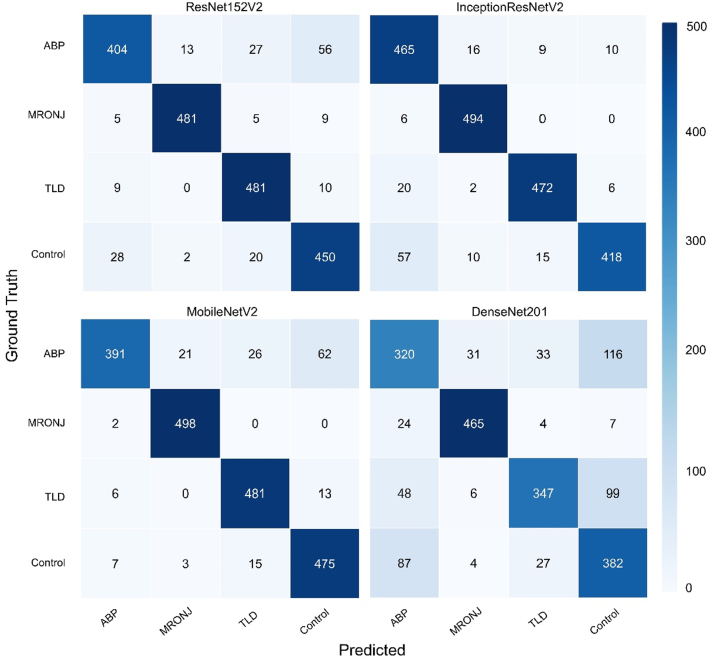
Multiclass confusion matrix of the test dataset (2000 images in total, 500 images for each class) for the four networks (InceptionResNetV2, ResNet152V2, DenseNet201, and MobileNetV2). The diagonal values refer to the correctly classified images (true positives), and the off-diagonal values depict misclassifications (false positives). Elements were colour-mapped according to the maximum and minimum values at the right colour-map bar.

Fig. 3 shows the ROC curves of each network for different classifications. The accuracy of classification, based on the area under the curves, was the highest in MobileNetV2 (mean 0.95), followed with a minor difference by InceptionResNetV2 (mean 0.947), ResNet152V2 (mean 0.937), and lastly, DenseNet201 (mean 0.84). Yet, InceptionResNetV2 had the best performance for the classification of ABP, while MobileNetV2 achieved the best performance when classifying MRONJ, TLD, and control groups. Additionally, the classification performance of the models was plotted through a confusion matrix (Fig. 4), comparing the class predictions against the ground truth.

**Fig. 5:**
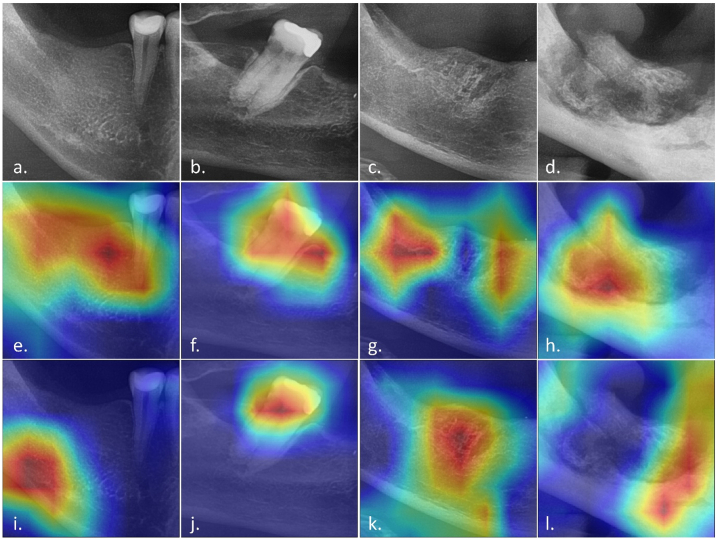
Gradient-weighted Class Activation Mapping (Grad-CAM) of the InceptionResNetV2 (e., f., g., h.) and the MobileNetV2 (i., j., k., l.) for a. control, b. thickened lamina dura (TLD), c. abnormal bone pattern (ABP), and d. osteonecrosis (MRONJ) images. The regions of interest for the algorithm are indicated by means of a warm and cold colour code. Being the warm regions, those in which greater attention was paid to the image features (high-weighted) and the cold regions in which there was less interest (low-weighted).

The heat maps of the two best performing networks, InceptionResNetV2 and MobileNetV2, are presented in Fig. 5 using a class activation map. Both networks assigned the highest activation regions to the distinguishing features of each trabecular bone pattern, displayed with warm colours in the figure. InceptionResNetV2 was more successful in combining the detection of globally and locally distributed features to discriminate the classes, while MobileNetV2 demonstrated a better performance in the detection of complex localised features.

**Fig. 6:**
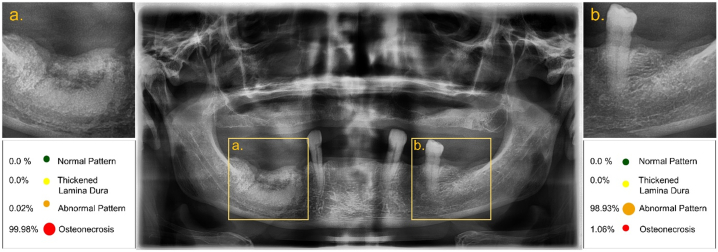
Display of the interface using a panoramic radiograph of a 70-year-old patient presenting with clinical bone exposure in the right posterior mandible. No other lesions were observed on the clinical examination. Once the image is imported, manual selection of different regions of interest (a, b) can be performed. The output will show the category to which the selected trabeculated region belongs together with the corresponding probability in the form of a colour-coded index.

## 4 Discussion

Convolutional neural networks have become a popular technique in the field of dentistry for detecting and classifying various pathologies and objects on radiographs (Estai et al., 2022a; Sivasundaram and Pandian, 2021; Sukegawa et al., 2021; Yang et al., 2020). Hence, we proposed in this study the use of CNNs for the automated classification of trabecular bone patterns in patients treated with antiresorptive drugs and who developed MRONJ. To the best of our knowledge, this is the first attempt to use an artificial intelligence model for this purpose.

All networks successfully classified all bone patterns and showed no statistical differences in their performance. From them, InceptionResNetV2 has already been used for dental applications, showing favourable results in automatic caries detection (accuracy of 0.87) (Estai et al., 2022b), and in the classification of mesiodens (accuracy 0.92) (Ahn et al., 2021) and teeth (accuracy 0.94) (Rajee and Mythili, 2021). Nevertheless, one of the main disadvantages of working with very deep networks like this one, is the need for computational power that is not always available in clinical settings and research laboratories (Sandler et al., 2018; Thompson et al., 2020). For this reason, we tested MobileNetV2, which, while maintaining its complexity, operates with fewer parameters and demands less sophisticated hardware (Sandler et al., 2018). Our results support the latter as a reliable substitute, as this network achieved the highest average AUC.

Despite achieving high accuracies, most of the misclassifications occurred between the abnormal bone pattern and the control images in all models. Perhaps an explanation lies in the distinctive features of each bone pattern. Most notable are the contrast differences. For instance, osteonecrosis lesions are bounded by clear radiolucent and radiopaque lines, resulting in high-contrast edges being easier to identify by both the human eye and the networks. On the other hand, abnormal patterns have less pronounced radiopaque areas distributed along the trabeculae which are less distinguishable from the homogeneous surface of the normal bone pattern. Given that the algorithms recognize high-contrast edges as seen in the Grad-CAM, images with normal and abnormal bone patterns become more challenging to classify.

Another explanation may be found in the images selected for each group. In the control group, the images were from a slightly younger population showing mostly sites with a natural dentition and with only 11 % of the ROIs involving fully edentulous areas. While 30 % of the ROIs in the abnormal bone pattern displayed such condition. When looking at the edentulous areas in the control group, InceptionResNetV2 misclassified 32 % of them into ABP. Furthermore, it was seen in the activation maps of several control images that teeth acted as a confounding factor with the area of interest resting on them, suggesting that the indicative feature of the control group was the presence of teeth and rather than the appearance of the bone.

Although there were no significant differences between the CNNs, InceptionResNetV2 was chosen for the development of the automated tool because of its better metrics and performance in ABP classification. While the other categories are important to recognize, they represent less of a challenge for the clinician and our interest lies in the early identification of sclerotic patterns as a risk factor for osteonecrosis (Moreno-Rabié et al., 2022). This CNN showed a superior performance in the detection of abnormal patterns due to the presence of large and small kernels at different equivalent depths in its architecture (Szegedy et al., 2016), allowing the efficient extraction and merging of globally and locally distributed features, such the mild radiopacities spread over the trabecular bone seen in these images. Additionally, to improve the performance of the model, an expert function could be added to the software where human-supervised corrections can be incorporated to learn from new data and rectify incorrect predictions.

Radiographic findings in patients treated with ARD are not uncommon (Moreno-Rabié et al., 2020). In this regard, the colour-coding system is of interest when these patients are to undergo tooth extraction, as it allows for easy diagnostic filtering. The presence of a green light or normal bone pattern will indicate a favourable scenario at that tooth extraction site, as this does not increase the likelihood of MRONJ (Arce et al., 2009; Moreno-Rabié et al., 2022; Nicolatou-Galitis et al., 2020). Furthermore, while the presence of thickening of the lamina dura is presented as a radiographic and pharmacokinetic marker indicating intake of these drugs (Klingelhöffer et al., 2016; Moreno-Rabié et al., 2022), bone changes related to antiresorptive drugs, such as osteosclerosis, have been associated with an increased risk for MRONJ (Arce et al., 2009; Moreno-Rabié et al., 2022; Nicolatou-Galitis et al., 2020). Finally, a red colour would indicate a settled osteonecrosis lesion in which referral to a specialized clinic for timely treatment is necessary.

Future applications of this diagnostic tool include evaluation prior to implant placement in patients treated with ARDs or early identification of MRONJ prior to bone exposure. It is worth noting that all regions of interest involving osteonecrosis belonged to mature lesions, which showed sequestrum formation, obvious lytic areas and osteosclerosis. Since the radiographic appearance of MRONJ is variable and does not necessarily correlate with clinical staging (Bedogni et al., 2012; Hamada et al., 2014), less obvious lesions should be presented to the network to assess possible differences between these and an abnormal bone pattern, given that mild radiographic osteonecrosis lesions are almost indistinguishable from sclerotic or abnormal bone patterns by the human eye. Consequently, some authors have suggested that these sites are latent osteonecrosis lesions that remain unexposed to the oral cavity (Bedogni et al., 2012; Moreno-Rabié et al., 2022; Nicolatou-Galitis et al., 2020; Saia et al., 2010).

Further studies should aim to overcome the limitations of this investigation. To prevent overfitting during training phase and improve the classification performance of the networks, the models should be less generalized by training them with a larger dataset (Yamashita et al., 2018). The dataset of this study was limited as it belonged to only one centre (Ahn et al., 2021) and given that MRONJ has a rather low incidence (Khan et al., 2015; Ruggiero et al., 2014). Although a novel augmentation method was used (Buslaev et al., 2020), a variety of data from different panoramic devices and with different scanning parameters are required to prevent biased classification. Moreover, the restricted hardware set up limited the employment of more trainable layers and increasing the batch size. Upgrading and utilizing a more powerful hardware would be imperative to improve the training results. Finally, the Grad-CAM visualisation showed that high contrast and sharp edges attract the highest attention of the models. Through employing feature selection, it would be possible to filter irrelevant or redundant features such as teeth, which are not involved in the classification of bone patterns. Hence, minimizing misclassification of the models.

## 5 Conclusion

In the present study, four different CNN architectures successfully classified different mandibular trabecular bone patterns showing reliable potentials for the identification of abnormalities in panoramic radiographs of antiresorptive treated patients. The best network, InceptionResNetV2, was selected for the development of a diagnostic tool. The proposed method is expected to support clinical decision making when alarming trabecular patterns are recognized, thereby minimizing complications with early diagnosis and treatment planning.

## CRediT authorship contribution statement

S.B.·S: formal analysis, methodology, writing original draft, writing-review & editing.

C.M.R: data curation, statistical analysis, writing original draft, writing-review & editing.

T.v.d.W: supervision, writing-review & editing.

R.J: conceptualization, supervision, writing-review & editing.

## Funding

Open access funding provided by Karolinska Institute.

## Declaration of competing interest

The authors declare that they have no known competing financial interests or personal relationships that could have appeared to influence the work reported in this paper.

## Data Availability

The data that has been used is confidential.
